# Sequential Treatment with Cytarabine and Decitabine Has an Increased Anti-Leukemia Effect Compared to Cytarabine Alone in Xenograft Models of Childhood Acute Myeloid Leukemia

**DOI:** 10.1371/journal.pone.0087475

**Published:** 2014-01-28

**Authors:** Sarah M. Leonard, Tracey Perry, Ciarán B. Woodman, Pamela Kearns

**Affiliations:** 1 School of Cancer Sciences, College of Medical and Dental Sciences, University of Birmingham, Edgbaston, Birmingham, United Kingdom; 2 Cancer Research UK Clinical Trials Unit, School of Cancer Sciences, University of Birmingham, Edgbaston, Birmingham, United Kingdom; University of Navarra, Spain

## Abstract

The current interest in epigenetic priming is underpinned by the belief that remodelling of the epigenetic landscape will sensitise tumours to subsequent therapy. In this pre-clinical study, paediatric AML cells expanded in culture and primary AML xenografts were treated with decitabine, a DNA demethylating agent, and cytarabine, a frontline cytotoxic agent used in the treatment of AML, either alone or in combination. Sequential treatment with decitabine and cytarabine was found to be more effective in reducing tumour burden than treatment with cytarabine alone suggesting that the sequential delivery of these agents may a have real clinical advantage in the treatment of paediatric AML. However we found no evidence to suggest that this outcome was dependent on priming with a hypomethylating agent, as the benefits observed were independent of the order in which these drugs were administered.

## Introduction

Survival rates for patients with acute myeloid leukemia (AML) remain inadequate with an overall survival of 40–45% reported in younger adults, and 63% in under 16 year olds who have a relapse rate of 35% [1]. While both anthracyclines and cytarabine (Ara-C) provide an effective backbone for most AML treatment protocols, new therapies offering a survival advantage over current standard treatments have been elusive, with the possible exception of the calicheamicin-conjugated antibody gemtuzumab ozogamicin [2]. Increasing interest in targeting epigenetic pathways has led to multiple studies of DNA demethylating agents, including decitabine (DAC) administered in low dose regimens [3]–[5]. DAC is a nucleoside analog believed to have multiple distinct mechanisms of action, including; activation of methylation-silenced tumor-suppressor genes, up-regulation of microRNA and induction of DNA damage responses [6]–[10]. DAC achieved marketing authorisation for the treatment of MDS (approved in the US, based on randomised study versus best supportive care) and for AML in older patients (approved in the EU, following randomised controlled study versus cytarabine or best supportive care) [3], [11], [12]. More recently, DAC has shown to be active in the treatment of very high risk relapsed or refractory AML in children [13]. Although studies in leukaemic cell lines have suggested additive effects from combining DAC and Ara-C, the potential benefit of adding DAC to the multi-agent treatment regimens that are standard care for AML in children and young adults has not been explored [14]. Using primary AML samples, we investigated the most effective scheduling of DAC and Ara-C, both *in vitro* and in primary AML xenografts and explored the epigenetic and transcriptional changes associated with their use.

## Methods

### Patient Ethics

Paediatric AML bone marrow (BM) cells were obtained from the Birmingham Children's Hospital with fully informed written consent from parents. This study was approved by the Multicentre Research Ethics Committee, Birmingham Children's Hospital, Childhood Cancer and Leukaemic Group (MREC number; CCLG08/H0405/22).

### Cell Culture

Following positive isolation using magnetic cell separation (Miltenyl Biotec Inc, Germany), 5×10^4^ CD34+ blasts were cultured on MS5 stromal cells and expanded in hematopoietic media (Myelocult/Stemcell, Grenoble, France) containing 1 mM hydrocortisone, IL-3, GCSF and TPO (20 ng/ml) (Peprotech, Rocky Hill, NJ, USA). Following weekly demi-depopulation, long-term cultures were established from 8 of 11 patient samples as previously described [15], [16].

### Drug Treatment

For growth inhibition assays, primary AML cells were plated at a density of 5×10^3^/mL in 1 mL medium 24 h before treatment. Cells were treated with serial dilutions of DAC (0.005–50 µM) or Ara-C (0.001–10 µM) at 0, 24, 48, 72 and 96 h. Cell viability was measured by trypan blue exclusion and cell proliferation using the CellTiter96 assay (Promega, Madison, WI, USA). When measuring the half maximal (50%) inhibitory concentration (IC50), fresh DAC was added every 24 h without changing the medium. The doses that inhibited proliferation to 50% (IC50) after 120 h of treatment were analysed using the median-effect method. In sequential studies using DAC and Ara-C, 5 AML cultures were treated with the first drug for 5 days followed by the second for 5 more. Cell cycle analysis was performed on treated cells stained with propidium iodide. DNA and RNA were extracted from cells using an Allprep kit (Qiagen, Valencia, CA, USA).

### Murine Studies

The Birmingham Biomedical Ethics Review Subcommittee (BERSC) approved all animal protocols in this study. *In vivo* experiments were performed on 6–8 week female NOD/Shi-scid/IL-2Rγnull (NOG) and were carried out in accordance with UK Home Office Guidelines. Three xenografts were established (36 mice per experiment), using primary paediatric AML patient samples with different cytogenetic profiles (AML–XG1- FLT3-ITD mutation with uniparental disomy on chromosome 13; AML–XG2 - MLL rearrangement; AML–XG3 - translocation 45, XX). 10–20 weeks following tail vein injection with 7.7-10×10^5^ bulk primary cells, the 36 animals were randomised using GraphPad Software random number generator into 6 groups of 6 mice. Animals were treated with vehicle (PBS), 0.5 mg/kg DAC or 75 mg/kg Ara-C by intraperitoneal injection either alone or in combination as set out in Table 1. For the final xenograft experiment, all animals were treated for 10 days so as to ensure that all groups had the same duration of treatment. Time to AML engraftment had previously been established for each sample by measuring levels of human CD45+/CD33+ cells in the BM from animals culled at intervals. Following 5 or 10 days of treatment, depending on the treatment regimen, animals were sacrificed and single cell suspensions were prepared from the spleen and BM. Flow cytometry using the BD LSRII was performed on these samples, which were stained for human CD45 and CD33 and for mouse CD45 (e-Bioscience, San Diego, CA, USA) and analysed using FACsDIVA software. The proportion of human cells (CD45+) which were CD45+CD33+ defined the level of leukaemic engraftment. Cells from the same treatment groups were pooled before magnetic isolation of either CD34+ or CD38+ cells (depending on the leukaemic subset present), and DNA and RNA extracted as before.

**Table 1:** Xenograft drug schedule.

Week 1 (5 days)	Week 2 (5 days)
PBS	
DAC alone 0.5 mg/kg	
Ara-C alone 75 mg/kg	
DAC 0.5 mg/kg with Ara-C 75 mg/kg (D+A)	
DAC alone 0.5 mg/kg	Ara-C alone 75 mg/kg (D/A)
Ara-C alone 75 mg/kg	DAC alone 0.5 mg/kg (A/D)

6 mice in each arm were administered DAC or Ara-C IP in the sequence and dose shown.

### Pyrosequencing Analysis

100 ng of DNA from primary AML cells treated for 5 days with or without DAC was bisulphite converted using the EZ DNA methylation kit (Zymo Research, Irvine, CA, USA). 20 ng of bisulphite modified DNA was used in each PCR and products run on a Qiagen pyromark system. Pyrosequencing primers were designed using PSQ primer design software (Qiagen). The PCR was performed in a total volume of 50 µl using 25 µl hotstart taq master mix (Thermo Scientific, Waltham, MA, USA), 5 pmol biotinylated primer, 10 pmol non-biotinylated primer and 10 µl bisulphite modified DNA. The pyrosequencing reactions were performed on a Pyromark ID system (Qiagen) and analysed using Pyro Q-CpG software (Qiagen).

### Quantitative PCR (Q-PCR)

mRNA levels following treatment were assayed using Q-PCR. cDNA was generated from 500 ng of xenograft RNA using the Superscript III First-strand synthesis system (Invitrogen, Carlsbad, CA, USA) with random primers (Promega). Q-PCR assays were prepared in a final volume of 25 µl which contained 1 µl cDNA, TaqMan universal PCR mastermix (Applied Biosystems, Foster City, CA, USA), B2M house-keeping assay (Applied Biosystems) and commercial Taqman assay for target genes; FLT3, MLL5, CTBP1, ILF3 and MARCKS. Q-PCR assays were performed in triplicate using an ABI Prism 7700 sequence detection system (Applied Biosystems). The 2-ΔΔ CT method was used to quantify expression relative to the housekeeping control.

### Methylation and Transcriptional Analysis

Infinium Illumina Methylation450 arrays were used to measure the difference in global DNA methylation between PBS and drug treated AML xenografts. 1 µg of control and drug treated DNA was bisulphite converted using the EZ DNA methylation kit (Zymo Research). Methylation data were normalized and background subtracted using Genome studio (Illumina, San Diego, CA, USA). Differentially methylated CpG sites were identified using Genome Studio; results were filtered to retain CpG in which the change in beta values between PBS and drug treated samples was >±0.2. Methylation data have been deposited in the Gene Expression Omnibus under accession number 44830.

Affymetrix U133 Plus 2 microarrays (Affymetrix, Santa Clara, CA, USA) were used to measure the difference in gene expression between PBS and drug treated AML xenografts. RNA quality was tested using a bioanalyzer and hybridized to microarray chips, which were analysed using the GCOS Software from Affymetrix, Inc. Probe level quantile normalization and robust multiarray analysis were performed using the Affymetrix package of the Bioconductor project. Differentially expressed genes were identified using Limma analysis with a fold-change threshold of 1.3. Transcriptional data have been deposited in the Gene Expression Omnibus under accession number 44857.

## Results

### Treatment of primary AML cultures

**Figure 1:**
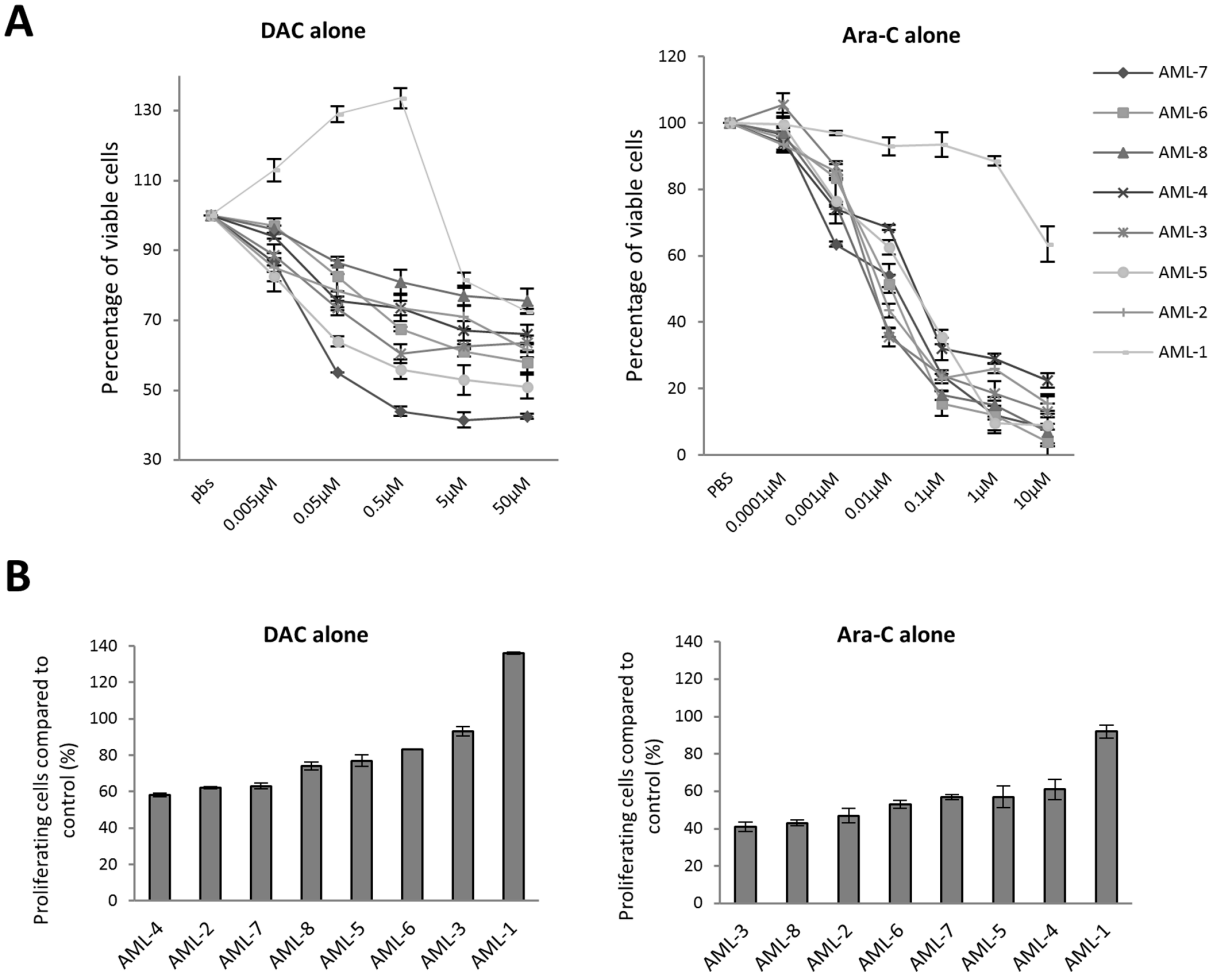
The *in vitro* effects of DAC and Ara-C in primary paediatric AML. A). Changes in viability following treatment with DAC or Ara-C at a range of concentrations in 8 primary AML patient samples. B) Changes in proliferation following treatment with DAC or Ara-C at the IC50 dose in 8 primary AML patient cultures. Cells were treated at 0, 24, 48, 72 and 96 h, both cell viability and proliferation were measured at 120 h. All experiments were performed in triplicate.

To compare the effect of DAC alone and in combination with Ara-C, 8 primary AML cultures were established *in vitro* and treated daily for 5 days with DAC and Ara-C either as single agents, in sequence or simultaneously. When administered as a single agent, Ara-C inhibited proliferation and reduced cell viability in a dose dependant manner in 7/8 primary AML cultures with IC50s ranging from 0.006 –0.04 µM. The remaining culture, AML-1, was relatively resistant to treatment with Ara-C (IC50 53 µM). Similarly, DAC also demonstrated cytotoxic and anti-proliferative activity against the same panel of primary AML cultures (IC50s 0.01 –0.06 µM, AML-1 4.5 µM) (Figure 1 and Table 2).

**Table 2:** IC50 dose following treatment with DAC and Ara-C alone.

	DAC IC50	Ara-C IC50
**AML-1**	4.5 µM	53 µM
**AML-2**	0.04 µM	0.008 µM
**AML-3**	0.03 µM	0.007 µM
**AML-4**	0.04 µM	0.03 µM
**AML-5**	0.02 µM	0.04 µM
**AML-6**	0.06 µM	0.01 µM
**AML-7**	0.01 µM	0.02 µM
**AML-8**	0.04 µM	0.006 µM

Cells were treated at 0, 24, 48, 72 and 96 h and the IC50 measured at 120 h.

**Figure 2:**
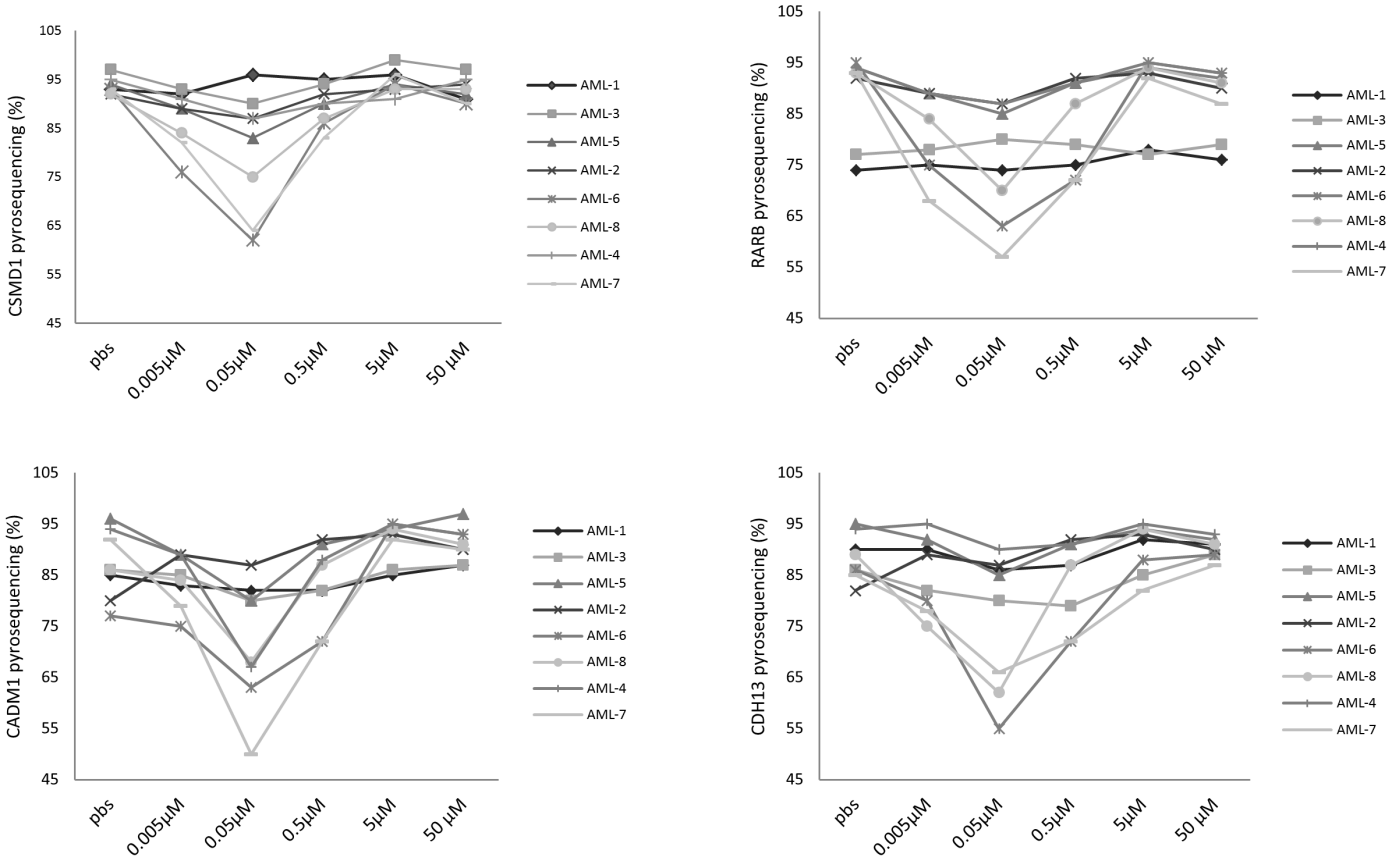
Pyrosequencing analysis of 4 tumour suppressor genes following DAC treatment. Changes in methylation status of a CpG site for 4 representative candidate genes (CSMD1, RARB, CADM1 and CDH13), following treatment with DAC at a range of concentrations in 8 primary AML patient cultures. Cells were treated at 0, 24, 48, 72 and 96 h, DNA methylation was measured at 120 h. All experiments were performed in duplicate.

When selecting the dose of DAC for use in combination with Ara-C, we aimed to identify that dose which achieved maximum demethylation in our panel of primary AML cultures. Using pyrosequencing, we examined the methylation of four candidate tumour suppressor genes, known to be hypermethylated in haematological malignancies. We found that in each primary culture, maximum demethylation was induced following treatment with 0.05 µM DAC; however, the level of demethylation achieved with this dose of DAC differed across the panel of AML samples, and did not correspond to the IC50 (Figure 2).

**Figure 3:**
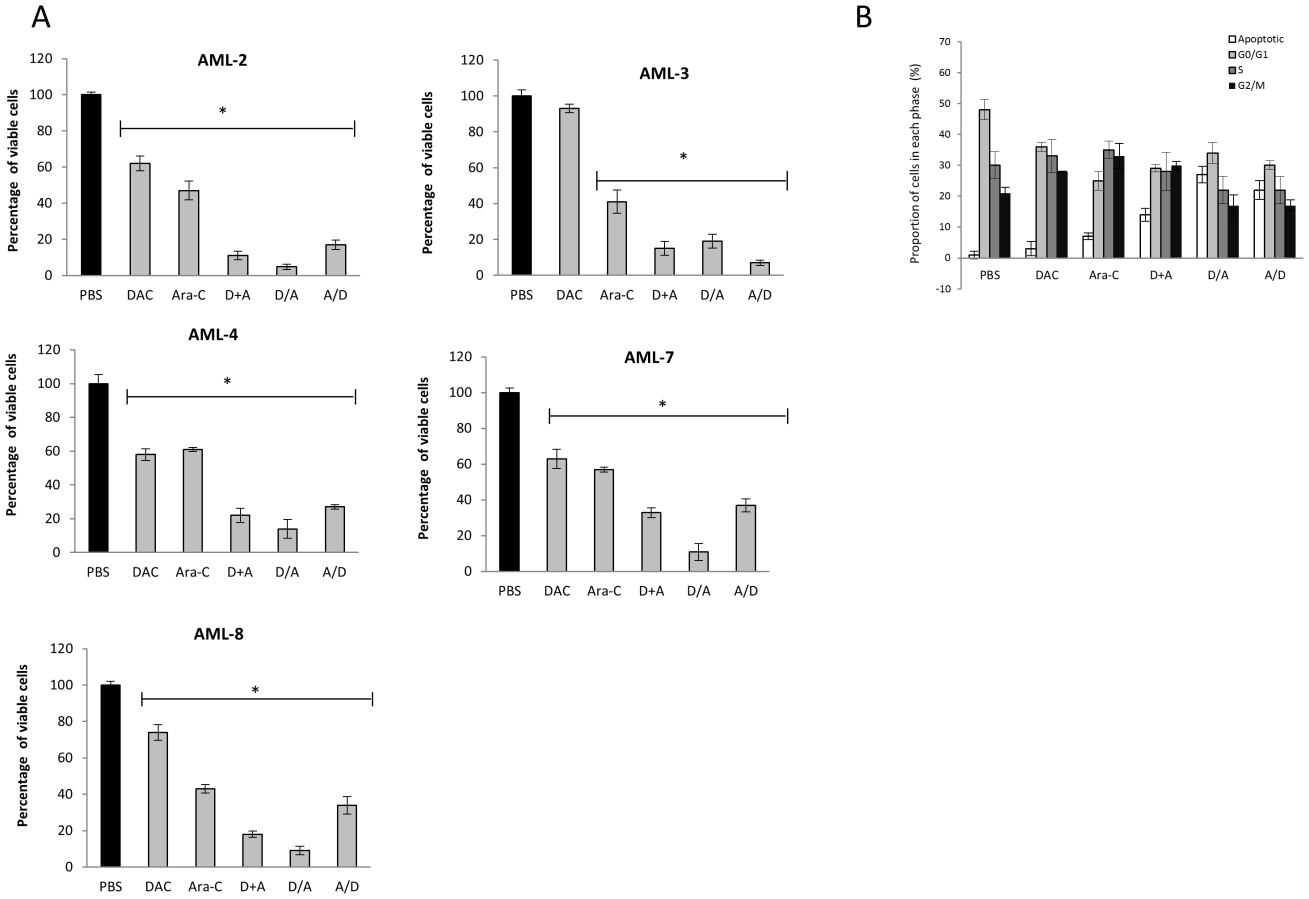
The *in vitro* effects of DAC and Ara-C in combination in primary paediatric AML. **A**) The percentage of viable cells following treatment with each drug regimen compared to PBS controls in 5 primary AML patient cultures (* P<0.01). All experiments were performed in triplicate. **B**) The percentage of cells in each phase of cell cycle following treatment with each drug regimen, for a representative primary AML sample (AML-7). All experiments were performed in duplicate.

We next explored the effect of low dose DAC treatment (0.05 µM) combined with the sample-specific IC50 dose of Ara-C, on 5/8 AML primary cultures for which sufficient cell number were available. We showed that both sequential and simultaneous administration of DAC and Ara-C induced a greater decrease in cell viability than either drug alone. When the drugs were administered sequentially, the decrease in viability was greatest when cells were treated with DAC for 5 days followed by Ara-C for 5 days in 4/5 samples tested. In the remaining sample, AML-3, the decrease was greater when Ara-C was followed by DAC (Figure 3A). Cell cycle analysis following treatment of primary AML cultures with DAC and Ara-C at the concentrations described above, revealed a decrease in the proportion of cells in G0/G1 phase and an increase in the proportion of cells in G2/M phase. Both sequential and simultaneous treatment resulted in a depletion of cells in G0/G1 phase and an increase in apoptotic cells; treatment with DAC followed by Ara-C caused the greatest increase in apoptosis (Figure 3B).

### Treatment of AML xenografts

**Figure 4:**
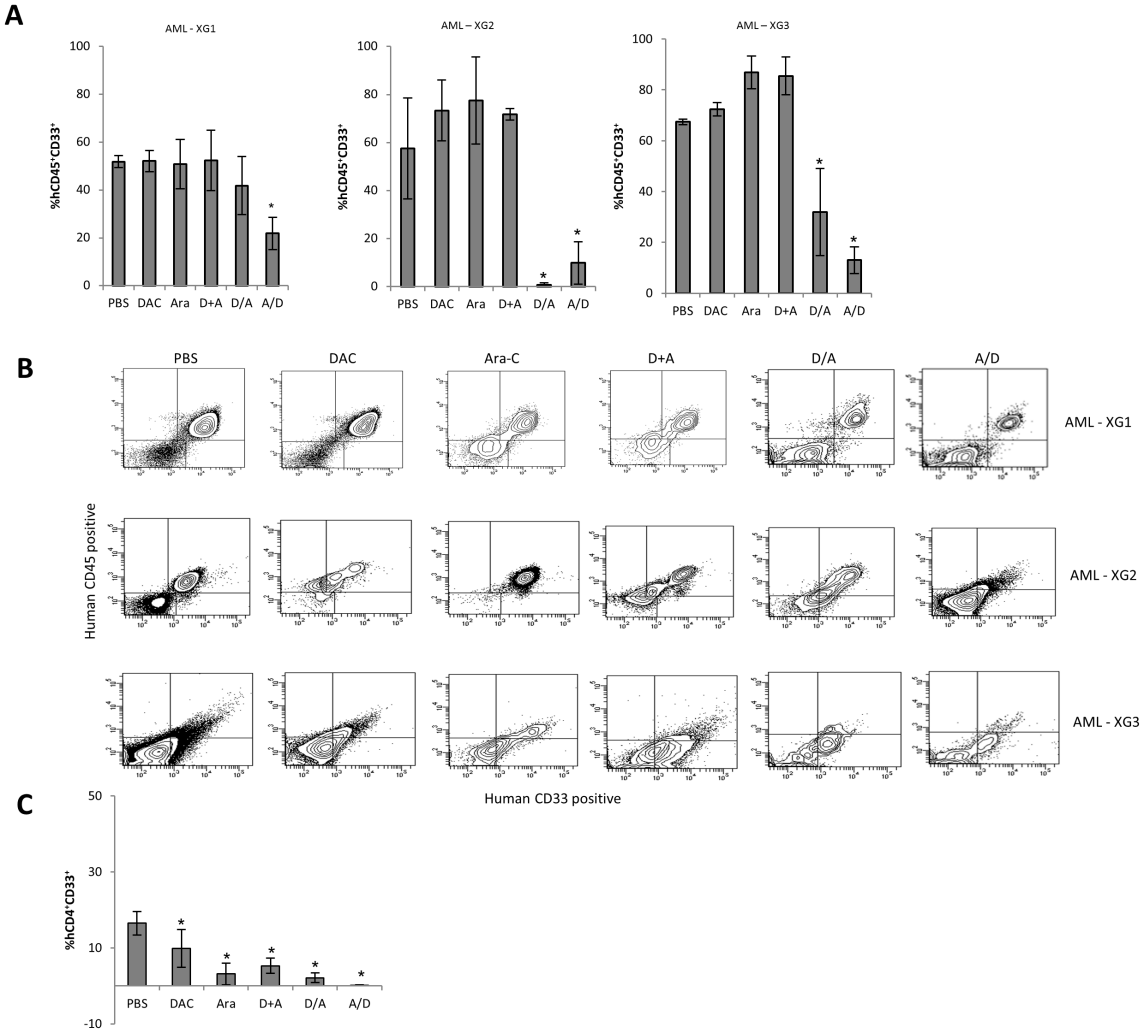
The in vivo effects of DAC and Ara-C in primary paediatric AML xenografts. A and B) Cells isolated from BM of three xenografts following 5 or 10 days of treatment were stained for human CD45 and CD33 and for mouse CD45; the proportion of human cells which were CD45^+^CD33^+^ from each treatment group were compared to Ara-C treatment alone in the BM. C) Cells isolated from the spleen which were human CD45^+^CD33^+^ compared to PBS (* P <0.001).

We next examined the impact of DAC and Ara-C alone or in combination, on human AML blast cell survival in three primary paediatric AML xenografts according to the schedules set out in Table 1. Two of these xenografts were established using those samples which had shown the greatest decrease in methylation following DAC treatment in vitro (AML-6, AML-XG1 and AML-7, AML-XG2). The remaining xenograft was established using a sample which had shown little methylation change when treated with DAC in primary culture but had shown the greatest change in cellular proliferation following DAC treatment in vitro (AML-4, AML-XG3). The endpoint of these experiments was the level of BM engraftment human CD45+/CD33+ at a fixed time point following one cycle of treatment. Notably, treatment with Ara-C or DAC alone or when administered simultaneously did not reduce the proportion of human CD45^+^/CD33^+^ BM engraftment compared to PBS controls in any of the three xenografts. In contrast, sequential administration of Ara-C and DAC induced a significant decrease in these cell populations when compared to both PBS controls and Ara-C alone (Figure 4A and B). It is conceivable that the decrease observed in the first two xenograft experiments (AML-XG1 and AML-XG2), was a result of sequential treatment being delivered for 10 days whereas the DAC and Ara-C alone groups were treated for 5 days. However, a significant decrease was observed in the final xenograft experiment when the DAC and Ara-C alone groups were also treated for 10 days. DAC and Ara-C injected as single agents and in combination were well tolerated following days of treatment, with <10% weight loss observed in those animals given a second week of treatment. In the single xenograft (AML-XG1) in which splenic AML engraftment was observed, the proportion of human CD45^+^/CD33^+^ cells in the spleen were significantly lower in all treatment groups when compared with controls (Figure 4C).

### Genome-wide expression and methylation profiling

**Figure 5:**
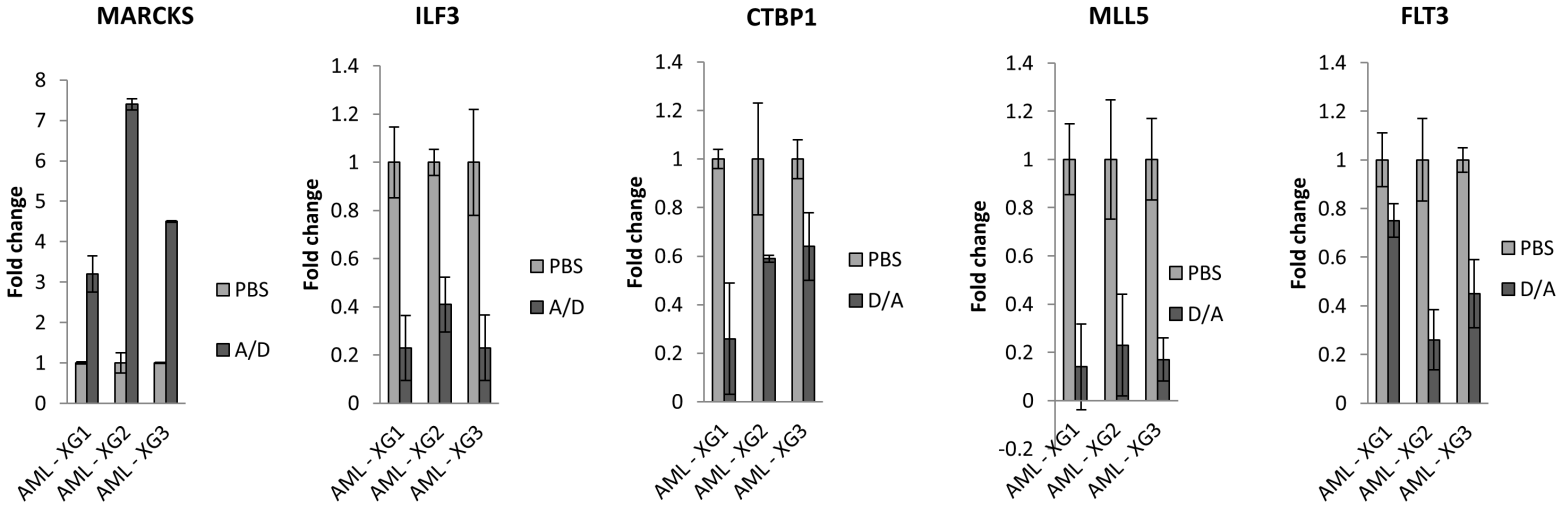
Q-PCR analysis of candidate genes identified from the expression arrays. Changes in the expression of 5 genes which were concordantly de-regulated in all three xenografts following sequential treatment with either Ara-C followed by DAC (ILF3 and MARCKS) or DAC followed by Ara-C (FLT3, CTBP1 and MLL5). Assays were carried out in triplicate.

DNA methylation and gene expression profiling were performed on AML blasts from all three xenografts following treatment with PBS, DAC, Ara-C, or both, delivered either sequentially or simultaneously. Pyrosequencing was used to validate methylation changes at selected CpG sites and Q-PCR used to validate changes in concordantly regulated candidate genes, some of which are known to be associated with reduced cell proliferation, invasion and survival in AML or other tumour types (Figure 5).

DNA methylation profiling revealed that less than 4% of all CpG sites were changed with DAC treatment alone, whereas approximately a third of all CpG sites were changed following either of the sequential treatments. The ratio of hypomethylation to hypermethylation changes also differed dependent on the drug treatment schedule. While DAC was almost exclusively a hypomethylating agent in all xenografts, treatment schedules that included Ara-C were associated with a greater proportion of hypermethylation changes. Interestingly, both hypermethylation and hypomethylation changes were less pronounced when Ara-C and DAC were given simultaneously (Table 3).

**Table 3:** Summary of methylation changes following treatment with different drug regimens.

AML – XG1	Total number of CpG sites changed	% of total CpG sites changed	Hypomethylated	Hypermethylated	Ratio of hypomethylated to hypermethylated changes	Median change in Beta Value
**PBS vs DAC**	17275	3.8	17258	17	1015∶1	−0.032
**PBS vs Ara-C**	122690	26.4	85702	36988	2∶1	0.025
**PBS vs D+A**	9906	2.0	5223	4683	1∶1	0.015
**PBS vs A/D**	164220	34.4	156585	7635	21∶1	−0.028
**PBS vs D/A**	162719	35.7	123987	38732	3∶1	−0.064
**AML - XG2**						
**PBS vs DAC**	14879	3.0	14857	22	675∶1	−0.042
**PBS vs Ara-C**	156544	33.3	118532	38012	3∶1	0.04
**PBS vs D+A**	12809	2.8	9611	3198	3∶1	0.0255
**PBS vs A/D**	111255	22.9	102419	8836	12∶1	−0.047
**PBS vs D/A**	170595	35.8	110686	59909	2∶1	−0.078
**AML - XG3**						
**PBS vs DAC**	10304	2.2	10290	14	735∶1	−0.0485
**PBS vs Ara-C**	137804	28.9	84792	53012	2∶1	0.011
**PBS vs D+A**	8831	1.9	3433	5398	1∶1	0.005
**PBS vs A/D**	99420	21.3	93227	6193	15∶1	−0.0195
**PBS vs D/A**	204784	43.2	143348	61436	2∶1	−0.092

For each xenograft, the number of hypomethylation and hypermethylation changes as well as the median change in beta value following treatment with each drug regimen. Differentially methylated CpG sites were identified using Genome Studio; results were filtered to retain CpG in which the change in beta values between PBS and drug treated samples was >±0.2.

Hypomethylation changes following treatment with any drug combination, were most common at CpG sites which had high beta values (high level methylation) in control-treated cells, and irrespective of the regimen used, hypomethylation occurred more commonly in gene bodies than in CpG sites closer to transcriptional start sites. In contrast, hypermethylation changes following treatment with any drug combination were most common at CpG sites which had low beta values (low level methylation) in control-treated cells and occurred more frequently at transcriptional start sites (Figure S1, Table S1). Although extensive DNA methylation changes were observed with all drug combinations tested, the overlap between the top 1000 concordantly changed CpG sites across the three xenografts was small (Figure S2).

Genome wide profiling revealed a substantial number of transcriptional changes following drug treatment of each xenograft (Table S2). However, for each drug treatment, the number of genes concordantly changed across the three xenografts was small and these common transcriptional changes were unique to that treatment (Figure S3, Table S3). However, within each xenograft there was a modest but significant overlap in the transcriptional changes induced following sequential treatments, Ara-C followed by DAC and DAC followed by Ara-C (Figure S4).

## Discussion

Our results support previous studies showing that low doses of DAC have both cytotoxic and anti-proliferative effects on primary AML samples in vitro and are associated with DNA demethylation [17]–[19]. Our in vitro and in vivo data are also consistent with clinical reports indicating that low dose DAC is an active agent in the treatment of AML [11], [13]. However, our most compelling observation is the substantially increased anti-leukemia effect when DAC is given sequentially with Ara-C compared to when DAC and Ara-C were given alone. This effect could not be attributed to the longer duration of the sequential treatment which was delivered for 10 days compared to the DAC and Ara-C alone groups which were treated for 5 days because a significant decrease was observed in the final xenograft experiment when the DAC and Ara-C alone groups were also treated for 10 days. We have shown using AML xenografts that low dose DAC when given sequentially with Ara-C, regardless of the schedule has a substantially increased anti-leukemia effect compared to Ara-C alone.

Consistent with recent reports we confirmed using genome-wide methylation profiling that DAC is an effective demethylating agent when used to treat AML cells and that demethylation is more likely to occur at CpG sites which are heavily methylated *ab initio* [17]. Although the number of CpG sites demethylated following treatment with Ara-C was 10 times greater than that observed following DAC we found that Ara-C, unlike DAC frequently induces hypermethylation changes which is consistent with previous reports of AraC's epigenetic modulating activity [20], [21]. Simultaneous treatment with Ara-C and DAC resulted in substantially fewer hypomethylation and hypermethylation changes than were seen with use of either drug alone whereas sequential treatment resulted in substantially more. Consistent with our finding that sequential treatment was found to be effective in reducing tumour burden irrespective of the order in which these agents were administered, there was a significant and substantial overlap in the transcriptional changes observed in the same xenograft following treatment with DAC followed by Ara-C and Ara-C followed by DAC. However, the absence of any methylation or transcriptional overlap between xenografts following treatment with these sequential regimens may reflect the variability in their cytogenetic profiles. Given our small sample size and the absence of a xenograft that did not respond to sequential treatment, we were unable to identify predictive markers of drug response.

While we were unable to provide a mechanistic explanation for the benefits of combined sequential treatment, we were able to show using genome-wide methylation and expression profiling that “epigenetic priming” which is often advanced as a justification for combining cytotoxic agents with a demethylating agent is an implausible explanation for the beneficial effects of combined therapy observed in this study. It is likely that these issues will only be adequately addressed in the context of a clinical trial in which samples are prospectively collected from a cohort of responding and non-responding patients. Our findings lend critical weight to the need for clinical studies evaluating the use of combined therapy in paediatric AML patients.

## Supporting Information

Figure S1 Change in methylation values upon treatment with different drug regimens, versus methylation in mock-treated samples, for a representative primary AML sample (AML-XG1).
(TIF)

Figure S2 Number of concordant changes in methylation following treatment with each drug regimen. Results for both hypermethylation and hypomethylation changes are shown. There was no overlap for CpG sites hypermethylated following treatment with DAC alone.
(TIF)

Figure S3 Number of concordantly regulated genes across the three xenografts following treatment with each drug regimen.
(TIF)

Figure S4 Concordantly regulated genes following sequential treatment within the same xenograft.
(TIF)

Table S1 Methylation changes across different regions of the genome following treatment with different drug regimens. The frequency with which methylation changes were found at different locations in a xenograft following treatment based on the Illumina gene annotation (TSS1500, TSS200, 5′ UTR, 1^st^ Exon, gene body and 3′UTR).
(TIF)

Table S2 Summary of transcriptional changes following treatment with different drug regimens. For each xenograft, the number of up-regulated and down-regulated genes is shown.
(TIF)

Table S3 List of genes concordantly regulated following treatment with each drug regimen.
(XLSX)
