# Supplementary figures and images for: Sequential Treatment with Cytarabine and Decitabine Has an Increased Anti-Leukemia Effect Compared to Cytarabine Alone in Xenograft Models of Childhood Acute Myeloid Leukemia
Source: PLoS One. 2014 Jan 28;9(1):e87475. doi: 10.1371/journal.pone.0087475 (PMC3905025; doi:10.1371/journal.pone.0087475)

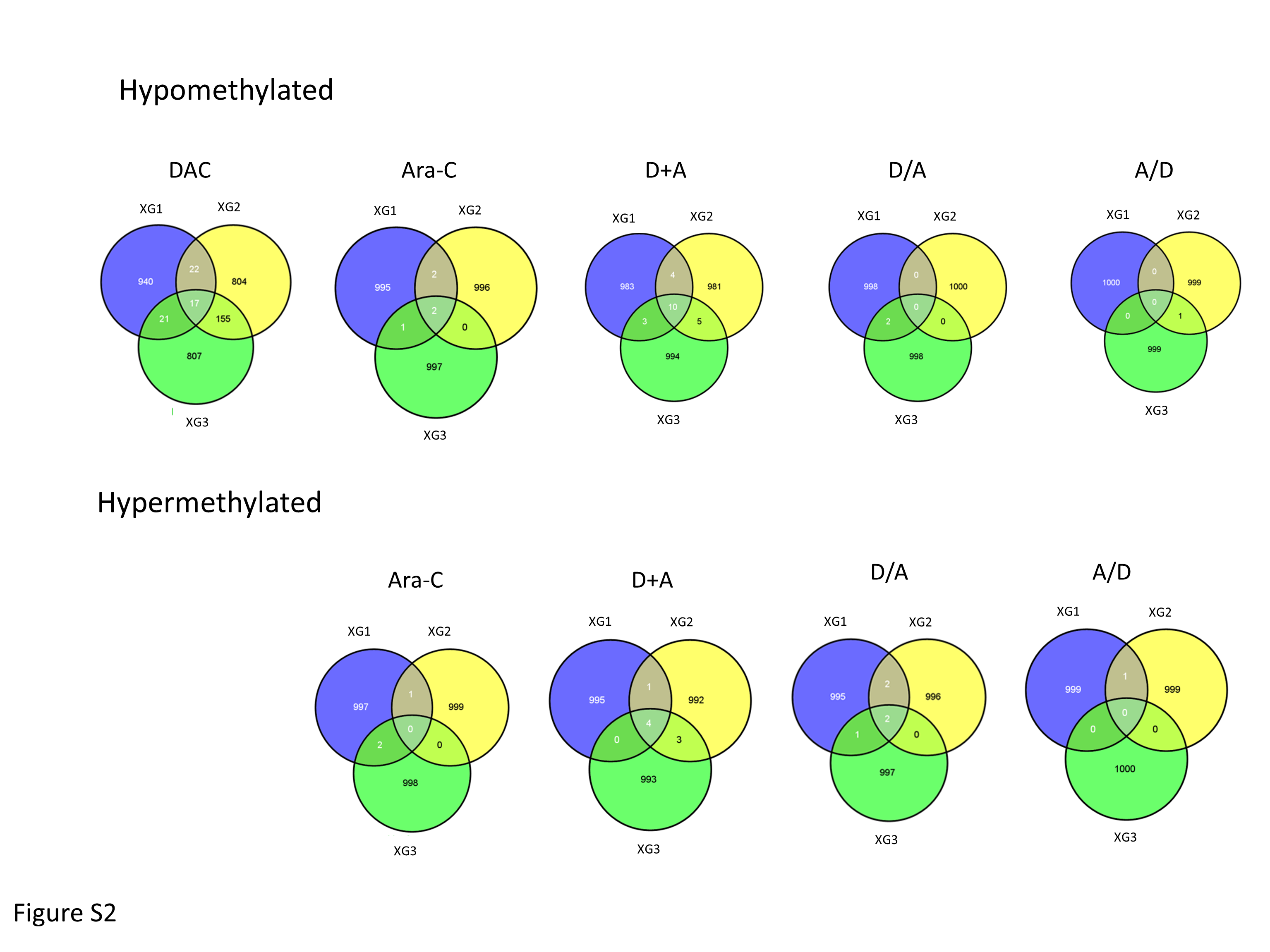

Supplement: Figure S2 — Number of concordant changes in methylation following treatment with each drug regimen. Results for both hypermethylation and hypomethylation changes are shown. There was no overlap for CpG sites hypermethylated following treatment with DAC alone. (TIF) [file pone.0087475.s002.tif]

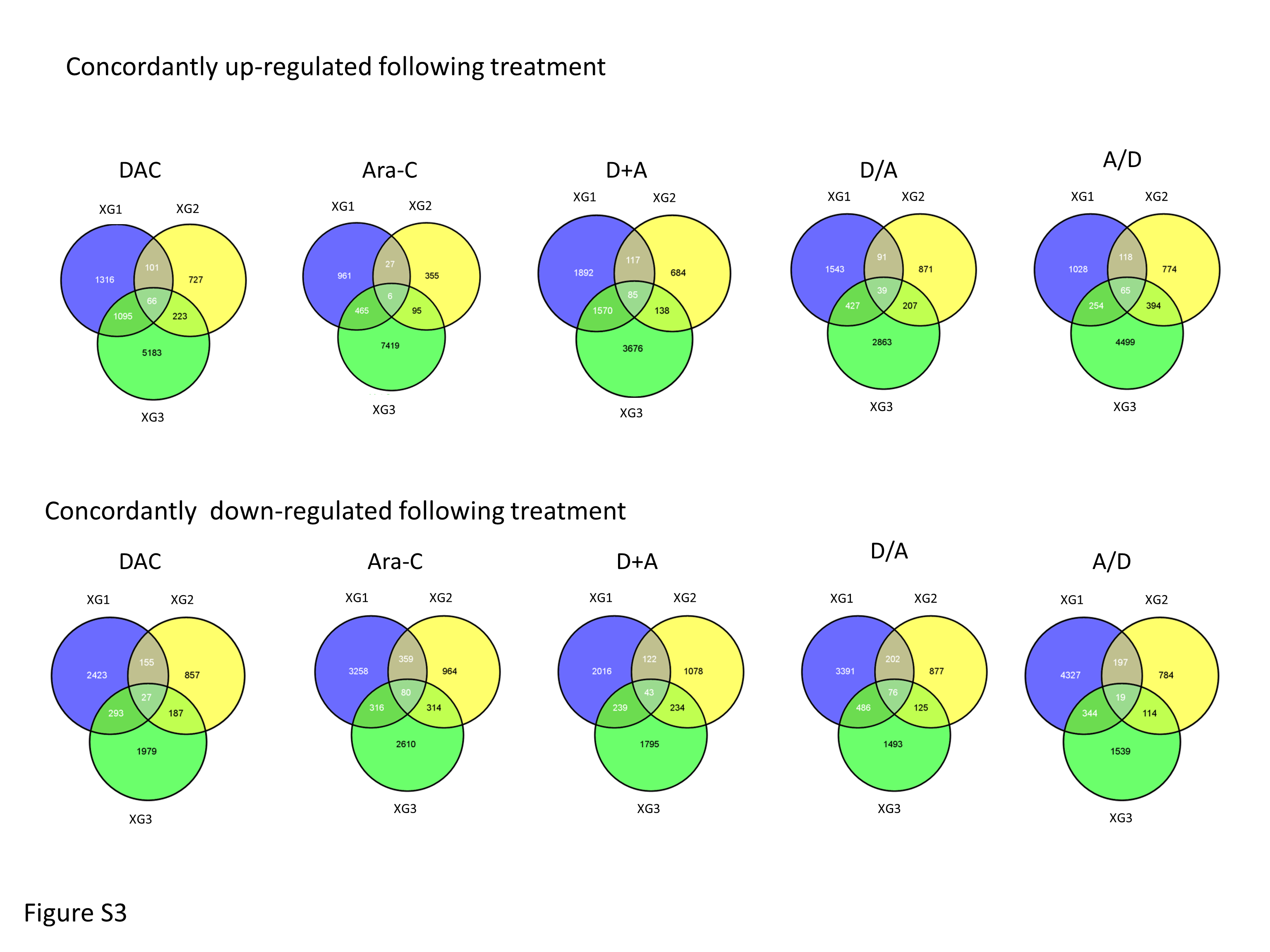

Supplement: Figure S3 — Number of concordantly regulated genes across the three xenografts following treatment with each drug regimen. (TIF) [file pone.0087475.s003.tif]

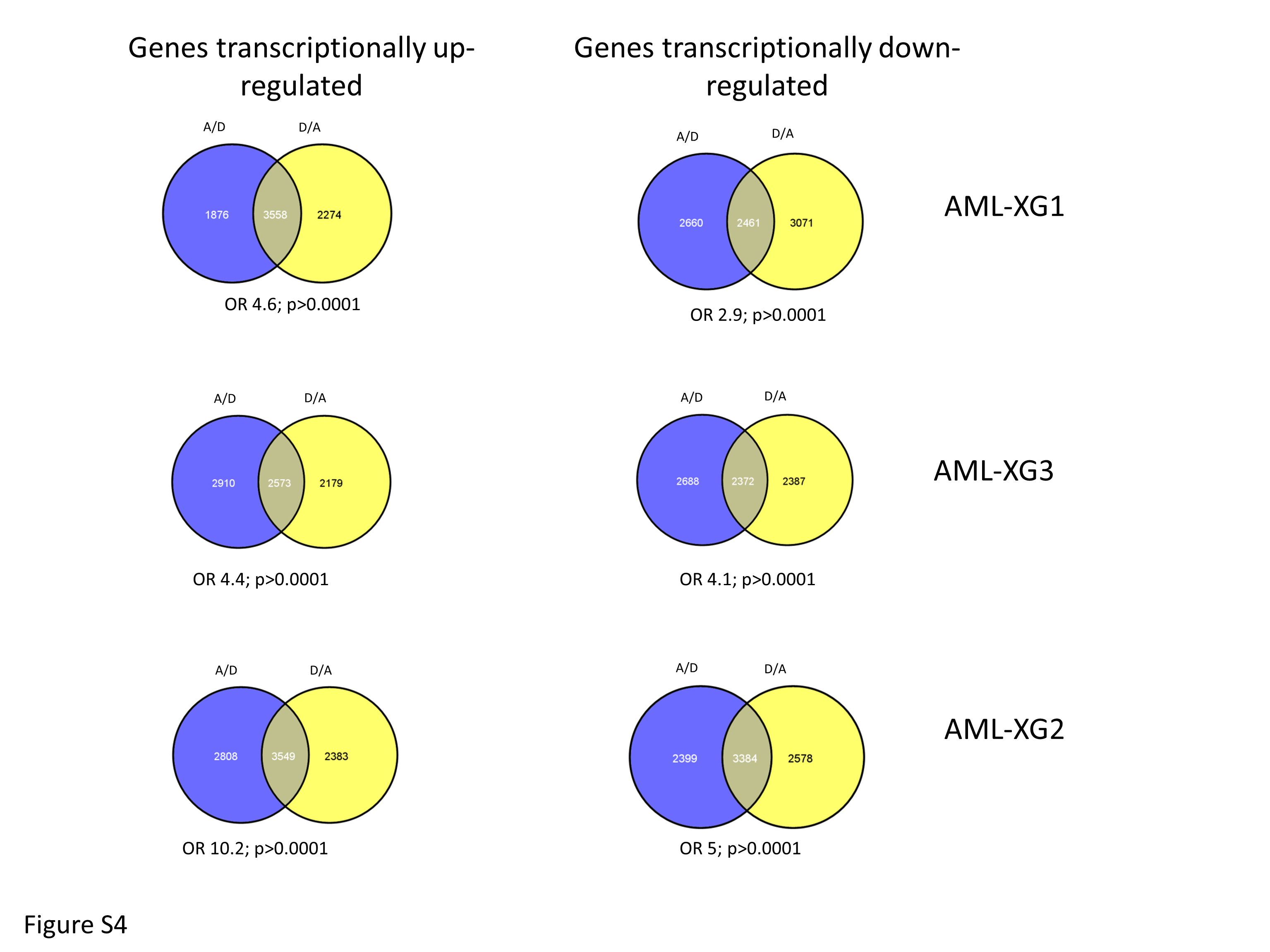

Supplement: Figure S4 — Concordantly regulated genes following sequential treatment within the same xenograft. (TIF) [file pone.0087475.s004.tif]

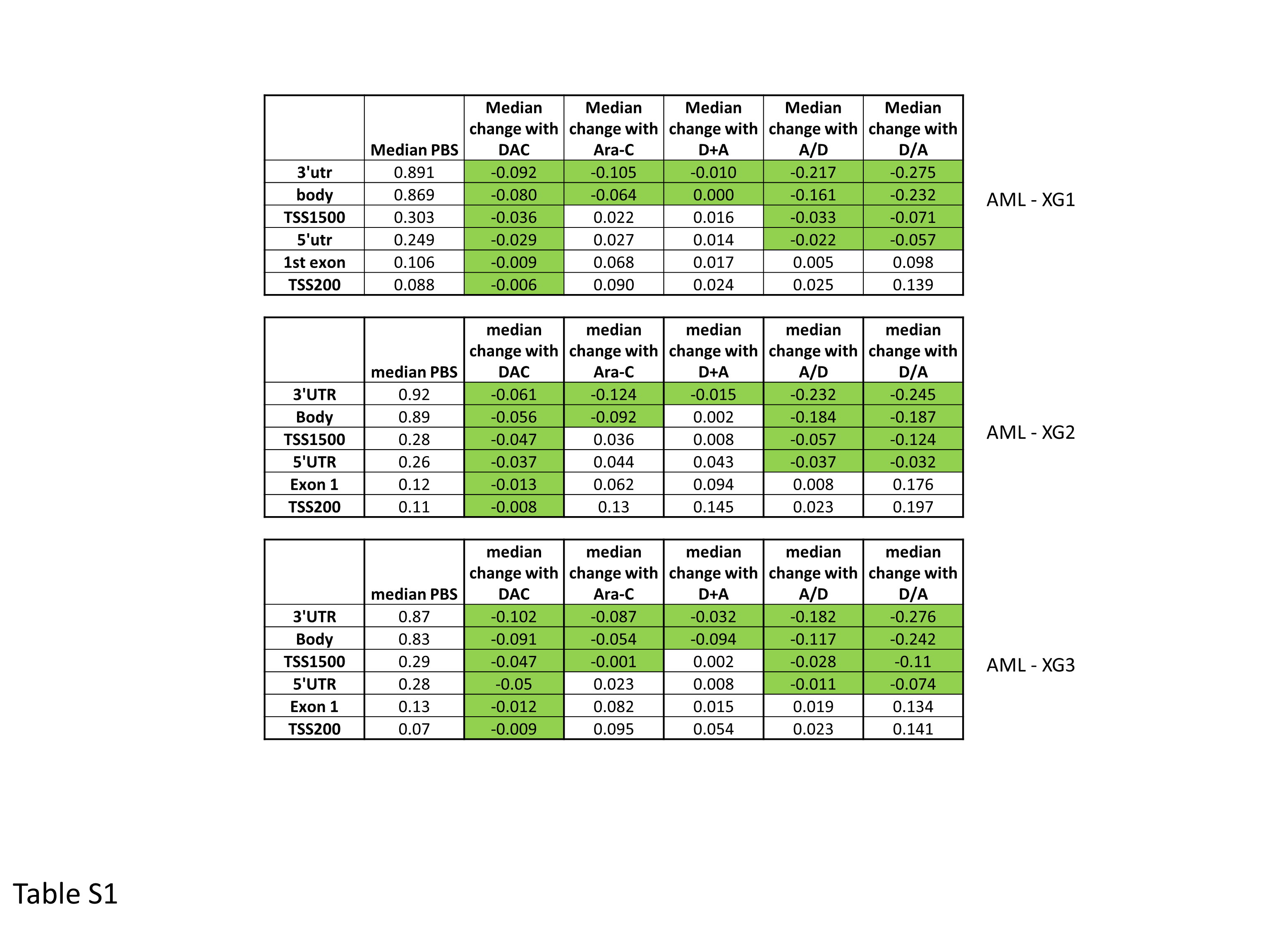

Supplement: Table S1 — Methylation changes across different regions of the genome following treatment with different drug regimens. The frequency with which methylation changes were found at different locations in a xenograft following treatment based on the Illumina gene annotation (TSS1500, TSS200, 5′ UTR, 1st Exon, gene body and 3′UTR). (TIF) [file pone.0087475.s005.tif]
